# Multiple Organ Dysfunction Syndrome in Malawian Children with Cerebral Malaria

**DOI:** 10.4269/ajtmh.24-0303

**Published:** 2024-10-08

**Authors:** Hunter J. Wynkoop, Alistair Bevan, Xochilt Galeano, Madiha Raees, Md Rejuan Haque, Terrie Taylor, Nicole F. O’Brien

**Affiliations:** ^1^ Division of Pediatric Critical Care Medicine, Department of Pediatrics, Nationwide Children’s Hospital, The Ohio State University, Columbus, Ohio;; ^2^ Chesterfield Royal Hospital NHS Foundation Trust, Derbyshire, United Kingdom;; ^3^ Division of Critical Care Medicine, Department of Pediatrics, Hospital Privado Salud Integral, Managua, Nicaragua;; ^4^ Division of Critical Care, Department of Anesthesiology and Critical Care, The Children’s Hospital of Philadelphia, Perelman School of Medicine at the University of Pennsylvania, Philadelphia, Pennsylvania;; ^5^ Department of Biomedical Informatics, Center for Biostatistics, The Ohio State University, Columbus, Ohio;; ^6^ Department of Osteopathic Medical Specialties, College of Osteopathic Medicine, Michigan State University, East Lansing, Michigan;; ^7^ Blantyre Malaria Project, Kamuzu University of Health Sciences, Blantyre, Malawi

## Abstract

More than 1,000 children under 5 years of age die every day from malaria. Cerebral malaria (CM) is the most severe and deadly manifestation of the disease. The occurrence of multiple organ dysfunction syndrome (MODS) has been associated with increased mortality in adult patients with CM. However, little is known about the frequency and severity of MODS in children with CM. This was a retrospective study of 199 pediatric patients with CM admitted to a referral hospital in Blantyre, Malawi, between January 2019 and May 2023. Data were abstracted from charts to calculate scores using four established scoring systems: Pediatric Logistic Organ Dysfunction-2 (PELOD-2), Pediatric Sequential Organ Failure Assessment (pSOFA), Signs of Inflammation in Children that Can Kill (SICK), and Lambaréné Organ Dysfunction Score (LODS). Mortality was 16% (*n* = 32). All four scoring systems were predictive of mortality, but the PELOD-2 and pSOFA scores outperformed the others with area under the curve values of 0.75 and 0.67, respectively. Multiple organ dysfunction syndrome was diagnosed in 182 patients (91%) using the PELOD-2 score, 172 patients (86%) using the pSOFA score, 99 patients (50%) using the SICK score, and 30 patients (15%) using the LODS. The PELOD-2 and pSOFA identify MODS in children with CM but require laboratory-based testing that is often unavailable in malaria-endemic areas. Furthermore, these scoring systems may identify primary malarial disease pathology rather than true organ dysfunction. Simplified scoring systems designed to recognize and quantify MODS in this patient population may provide opportunities for improved resource allocation and timely, organ-specific treatment.

## INTRODUCTION

Malaria is a debilitating disease that remains a significant public health challenge worldwide. An estimated 608,000 deaths occurred in 2022, with a disproportionate number of these fatalities occurring in sub-Saharan Africa.^1^ Mortality rates have remained relatively stagnant since 2015 despite the implementation of diverse preventive measures and the widespread use of artesunate. African children under 5 years of age continue to account for 76% of all malaria-related deaths.^1^^,^^2^ Cerebral malaria (CM), the most severe manifestation of the *Plasmodium falciparum* infection, has a mortality rate as high as 40% in African children.^3^^–^^8^

Multiple organ dysfunction syndrome (MODS), defined as the failure of two or more organ systems, is a complication of many critical illnesses including malaria. Multiple organ dysfunction syndrome has been identified in up to 85% of adult patients presenting with severe malaria.^9^ In adults with CM, the mortality rate increases from 8% in those with “pure CM” to 50% when MODS is also present.^9^^,^^10^ Despite the frequency and significant effects of MODS in adults with malarial infection, the presence of MODS and its impact on outcomes remain poorly understood in children with CM.

Historically, *P. falciparum* infection in African children was thought to have a “monosyndromic presentation” with minimal complications apart from the presenting coma, anemia, or acidosis.^11^ With the ongoing improvement of laboratory availability and monitoring, there is a growing recognition of the overlap in disease complications, such as a child admitted to the hospital with both CM and severe malarial anemia.^11^^,^^12^ In addition, it is plausible that multiple organs are simultaneously affected owing to the presence of parasitized red blood cells throughout the host’s microvasculature.^10^^–^^14^ Postmortem histopathological examinations of Malawian children with CM revealed sequestration of *P. falciparum* parasites within the brain, heart, lungs, spleen, stomach, intestines, and skin.^13^^,^^14^ Namazzi et al.^15^ evaluated 600 Ugandan children with malaria and reported increased mortality rates in patients diagnosed with both CM and acute kidney injury (AKI). These children with coma and AKI would meet the criteria for MODS. Within the same cohort, increased mortality was observed in patients with AKI and signs of cardiovascular compromise, another presentation of MODS. Improved understanding of the prevalence, severity, and overall impact of MODS in children with CM could have profound implications for clinical management and lead to better outcomes for affected children.

Multiple organ dysfunction syndrome can be diagnosed and quantified in pediatric patients using various scoring systems, including Pediatric Logistic Organ Dysfunction-2 (PELOD-2), Pediatric Sequential Organ Failure Assessment (pSOFA), Signs of Inflammation in Children that Can Kill (SICK), and Lambaréné Organ Dysfunction Score (LODS).^16^^–^^25^ The PELOD-2 and pSOFA scores are commonly used in pediatric intensive care units worldwide and have been validated to quantify MODS and severity of illness.^18^^–^^21^ Our group previously used the PELOD-2 scoring system to elucidate the frequency of MODS in 145 children with CM and identified MODS in 94% of cases.^26^ In adults with malaria, the SOFA score is used most frequently for diagnosing and quantifying MODS.^27^^–^^29^ To our knowledge, the pSOFA score has not been applied to pediatric patients with malaria. Both the PELOD-2 and pSOFA scoring systems rely heavily on laboratory-based values that are oftentimes not present in resource-limited settings. Contrarily, the SICK score was developed to predict mortality in children with infections in low-resource settings and is based on physical examination and vital sign abnormalities only.^22^^,^^23^ The LODS is a malaria-specific scoring system that aims to rapidly predict mortality in African children with this disease.^24^^,^^25^ No evaluation of the SICK or LODS scoring system to identify MODS has been undertaken. Our study aimed to evaluate the ability of these four scoring systems to recognize MODS and predict mortality in children with CM.

## MATERIALS AND METHODS

This was a retrospective review of prospectively collected data at Queen Elizabeth Central Hospital in Blantyre, Malawi, from January 2019 to May 2023. Our work was an ancillary study to Treating Brain Swelling in Pediatric Cerebral Malaria (5U01AI126610-02), an interventional, randomized clinical trial. Ethics approval was received through the Institutional Review Board at Michigan State University and locally through the University of Malawi College of Medicine Research Ethics Committee. Children between 6 months and 12 years of age who met the WHO’s case definition of CM (Blantyre coma score [BCS] of ≤2, peripheral parasitemia with *P. falciparum*, and no other detectable cause of encephalopathy) were eligible. Exclusion criteria included advanced HIV, gross malnutrition, and recent head trauma.

Patient data obtained on admission were extracted from paper charts, including vital signs, physical exam findings, and laboratory values. Laboratory studies included finger prick samples to ascertain the parasite species, packed cell volume, blood glucose concentration, lactate levels (Arkray Lactate Pro 2, Minneapolis, MN), creatinine (StatSensor Creatinine Meter, Nova Biomedical, Waltham, MA), total bilirubin (UNISTAT Bilirubinometer, Reichert Technologies, Depew, NY), and blood gas analysis (Abbott iSTAT, Princeton, NJ). Venous blood samples were collected for a full blood count and electrolyte analysis (Beckman Coulter Life Sciences Coulter Counter, Indianapolis, IN).

All patients in this study received treatment of severe malaria according to the Malawi Standard Treatment Guidelines. Management included the administration of intravenous artesunate; treatment of fever, seizures, and hypoglycemia; and the use of antibiotics as clinically indicated.^30^ None of the patients were receiving inotropic agents or mechanical ventilation upon admission.

To diagnose and quantify MODS, the data from each patient were scored using the following scoring systems: PELOD-2, pSOFA, SICK, and LODS. Minor adaptations were necessary, and the adapted scoring systems are included in Supplemental Tables 1–4. Notably, the Glasgow coma score (GCS) was not used in any scoring system because it was not recorded. Per standard of care in this patient population, the BCS was recorded and therefore used instead.^31^ For the neurologic assessment in PELOD-2 and pSOFA, we converted each score’s validated GCS cutoffs to clinically comparable BCS values. Similarly for the SICK score, the BCS was changed to the appropriate Alert, Voice, Pain, Unresponsive (AVPU) grade. To calculate the PELOD-2 score, creatinine values were adjusted from µmmol/L to mg/dL, and the SpO_2_/FiO_2_ ratio was used instead of PaO_2_/FiO_2_ owing to the complete absence of arterial blood gas values. All missing values were scored as normal for all four scoring systems.

For each patient, the number of affected organs was noted along with the specific organ systems involved. The SICK score and LODS lack predefined, organ-specific categories, necessitating the grouping of measured parameters by organ system (Supplemental Tables 3 and 4). For the SICK score, variables were organized into three organ-specific categories: neurologic (AVPU assessment), cardiovascular (capillary refill time, blood pressure, heart rate measurements), and respiratory (respiratory rate, oxygen saturation evaluations). Temperature, although a parameter in the SICK score, was not included in the number of affected organs as it does not correspond to a specific organ group. For LODS, parameters were grouped into two organ-specific categories: neurologic (coma, prostration) and respiratory (deep breathing).

Values are reported as median (interquartile range), mean (SD), or *n* (%) as appropriate. Receiver operator characteristic (ROC) curve analysis was used to assess the performance of each score to predict mortality. The sensitivity, specificity, positive predictive value, and negative predictive value of each scoring system were determined using a leave-one-out cross-validation analysis. For this cross-validation, we created training data removing one subject from the dataset. Next, we fit the model using the training dataset and subsequently used the fitted model to compute the optimal cutoff probability, maximizing sensitivity and specificity together. Finally, we used the data of the left-out subject as test data and estimated the outcome using the test data and the optimal cutoff probability from the model using the training data. We repeated this process for all the subjects in the dataset. Comparisons between survivors and nonsurvivors were analyzed using Fisher’s exact test, Mann–Whitney test, or Wilcoxon rank-sum test where appropriate. *P*-values <0.05 were considered statistically significant throughout. Analyses were conducted using GraphPad Prism v. 9.00 for Windows (GraphPad Software, La Jolla, CA).

## RESULTS

One hundred ninety-nine consecutive patients admitted between January 2019 and May 2023 were reviewed. Table 1 provides an overview of the cohort’s basic demographics, vital signs, and admission laboratory values. The mortality rate was 16% (*n* = 32).

**Table 1.** Demographics, laboratory investigations, and outcomes for the cohort

Variable	All (*N* = 199)
Age, Months, Median [IQR]	51 [33–75]
Male Sex, *n* (%)	96 (48)
Hours Since Fever Began, Median [IQR]	60 [48–72]
Hours of Unconscious State Prior to Admission, Median [IQR]	18 [9–24]
Had Seizures Prior to Admission, *n* (%)	124 (62)
Temperature, °C, Median [IQR]	38.5 [37.9–39.3]
Respiratory Rate, Breaths/Minute, Median [IQR]	36 [28–42]
Oxygen Saturation, %, Median [IQR]	97 [95–98]
Heart Rate, Beats/Minute, Median [IQR]	142 [127–158]
Mean Arterial Pressure, mm Hg, Median [IQR]	77 [70–87]
Pallor Present, *n* (%)	78 (39)
Jaundice Present, *n* (%)	10 (5)
On Mechanical Ventilation, *n* (%)	0 (0)
Retinopathy Positive, *n* (%)	119 (60)
Blantyre Coma Score, *n* (%)	
0	23 (12)
1	69 (35)
2	107 (54)
White Blood Cells, ×10^3^/*µ*L, Median [IQR]	9.4 [7.0–13.8]
Packed Cell Volume, %, Median [IQR]	26 [21–30]
Platelets, ×10^3^/*µ*L, Median [IQR]	73 [38–127]
Creatinine, mg/dL, Median [IQR]	0.7 [0.54–0.99]
Total Bilirubin, mg/dL, Median [IQR]	1 [0.3–1.8]
Lactate, mmol/L, Median [IQR]	4.1 [2.6–7.4]
Glucose, mg/dL, Median [IQR]	99 [81–121]
Mortality, *n* (%)	32 (16)

IQR = interquartile range.

**Figure 1.**
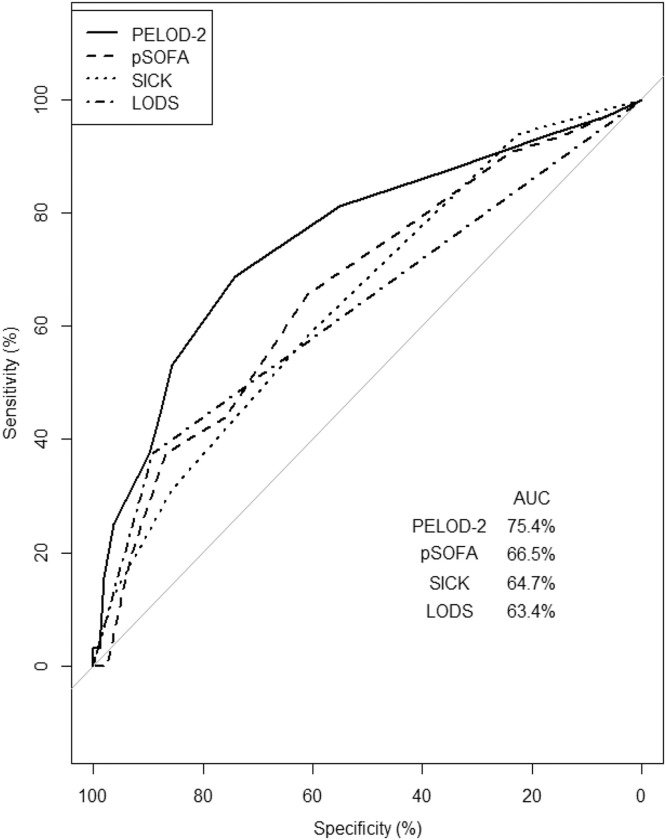
Receiver operator characteristic curve illustrating the ability of four different scoring systems to predict mortality. LODS = Lambaréné Organ Dysfunction Score; PELOD-2 = Pediatric Logistic Organ Dysfunction-2; pSOFA = Pediatric Sequential Organ Failure Assessment; SICK = Signs of Inflammation in Children that Can Kill.

When calculated based on admission data, a higher score on each of the four scoring systems was positively correlated with the risk of death. For every unit increase in the PELOD-2 score, the estimated odds of death increased by 36%. The odds of death increased by 29% for every unit increase in the pSOFA score and 64% for every point scored in the SICK score. All patients with CM had a minimum LODS of 2 based on their CM diagnosis. When the LODS increased to 3, the odds of death increased by 397%. When model performance to predict mortality was further evaluated, the PELOD-2 score outperformed all other models with an area under the curve value of 0.75. The ROC curves for the four scoring systems are illustrated in Figure 1. Further information regarding the sensitivity, specificity, positive predictive value, and negative predictive value of each scoring system can be found in Table 2.

**Table 2:** Results of leave-one-out cross-validation using the test datasets

Characteristic	PELOD-2 (*N* = 199)*	pSOFA (*N* = 199)*	SICK (*N* = 199)*	LODS (*N* = 199)*
Sensitivity	0.69 (0.5–0.84)	0.66 (0.47–0.81)	0.59 (0.41–0.76)	0.38 (0.21–0.56)
Specificity	0.74 (0.67–0.81)	0.61 (0.53–0.69)	0.60 (0.52–0.67)	0.89 (0.84–0.93)
Positive Predictive Value	0.34 (0.23–0.47)	0.24 (0.16–0.35)	0.22 (0.14–0.32)	0.40 (0.23–0.59)
Negative Predictive Value	0.93 (0.87–0.96)	0.90 (0.83–0.95)	0.88 (0.81–0.94)	0.88 (0.82–0.93)

LODS = Lambaréné Organ Dysfunction Score; PELOD-2 = Pediatric Logistic Organ Dysfunction-2; pSOFA = Pediatric Sequential Organ Failure Assessment; SICK = Signs of Inflammation in Children that Can Kill.

* Mean (95% CI).

**Figure 2.**
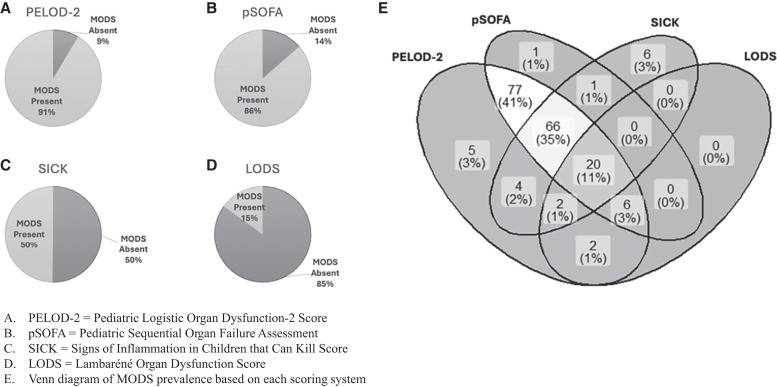
MODS prevalence using four different scoring systems in 199 children with CM. CM = cerebral malaria; MODS = multiple organ dysfunction syndrome.

**Figure 3.**
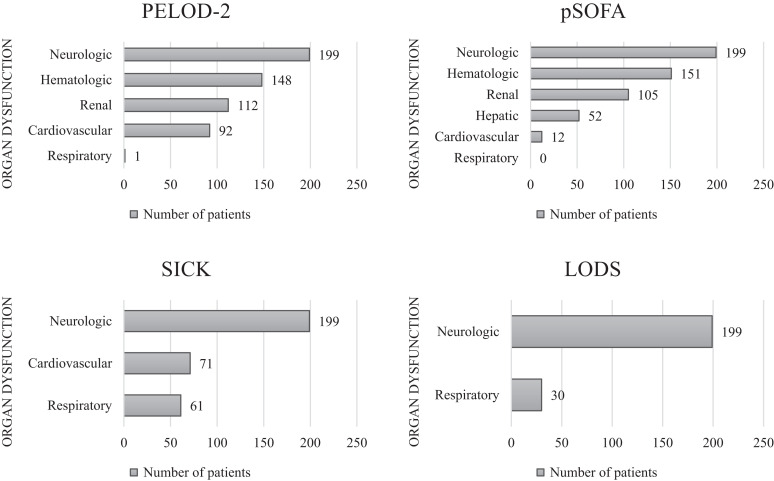
Individual organ dysfunctions by scoring system for the cohort. LODS = Lambaréné Organ Dysfunction Score; PELOD-2 = Pediatric Logistic Organ Dysfunction-2; pSOFA = Pediatric Sequential Organ Failure Assessment; SICK = Signs of Inflammation in Children that Can Kill. *N* = 199 patients.

Multiple organ dysfunction syndrome was identified in 190 of 199 patients (95%) in this cohort. The PELOD-2 score and pSOFA score revealed the presence of MODS in 91% and 86% of patients, respectively (Figure 2). Both scores indicated neurologic dysfunction in all 199 patients. The hematologic and renal systems were the second- and third-most affected organ systems based on the PELOD-2 and pSOFA scores (Figure 3). Moreover, children with more organs involved had higher rates of mortality (Table 3). When evaluating for MODS using the SICK score and LODS, 50% of patients and 15% of patients, respectively, met our predefined criteria.

**Table 3:** Organ dysfunction and mortality

Evaluation of Organ Dysfunction	All (*N* = 199)	Survivors (*n* = 167)	Nonsurvivors (*n* = 32)	*P*-Value
Organ Dysfunction Scores				
PELOD-2, median [IQR]	5.00 [3.00–6.00]	4.00 [3.00–6.00]	7.00 [5.00–9.25]	<0.001
pSOFA, median [IQR]	6.00 [5.00–8.00]	6.00 [5.00–7.00]	7.00 [6.00–9.00]	0.003
SICK, median [IQR]	2.00 [2.00–3.00]	2.00 [2.00–3.00]	3.00 [2.00–4.00]	0.006
LODS, *n* (%)				
2	169 (85)	149 (89)	20 (63)	<0.001
3	30 (15)	18 (11)	12 (38)	
Number of organ dysfunctions using PELOD-2, median [IQR]	3.00 [2.00–3.00]	3.00 [2.00–3.00]	3.00 [2.00–4.00]	0.059
Number of organ dysfunctions using pSOFA, median [IQR]	3.00 [2.00–3.00]	3.00 [2.00–3.00]	3.00 [2.00–3.00]	0.328

IQR = interquartile range; LODS = Lambaréné Organ Dysfunction Score; PELOD-2 = Pediatric Logistic Organ Dysfunction-2; pSOFA = Pediatric Sequential Organ Failure Assessment; SICK = Signs of Inflammation in Children that Can Kill.

## DISCUSSION

Multiple organ dysfunction syndrome is a major cause of death in critically ill children.^32^^–^^38^ Furthermore, mortality continues to escalate as the severity of organ dysfunction increases and as the number of organ systems involved increases.^33^^–^^40^ Despite the belief that MODS is a complication only seen in adults with severe malaria, our group has previously demonstrated both the presence of MODS in pediatric patients with CM and its association with increased mortality rates.^11^^,^^26^^,^^41^ This current study augments our previous findings of MODS prevalence in children with CM by applying a second validated organ dysfunction scoring system as well as two scoring systems specifically developed for use in resource-limited areas. In addition, our findings align with rates of observed MODS in adults with severe malaria.^27^^,^^28^

The PELOD-2 scores and the pSOFA scores revealed comparable rates of MODS within our cohort upon admission. One notable difference in the results of these two scoring systems was the presence of cardiovascular dysfunction, which was identified in more patients when using the PELOD-2 score (92 patients) compared with the pSOFA score (12 patients). This variance may be attributable to the use of lactate as one of the two markers of cardiovascular dysfunction in the PELOD-2 score. Although elevated lactate can indicate cardiovascular dysfunction and shock, multiple other factors are more likely causative in children with CM. For example, tissue hypoxia often results from profound anemia and microvascular occlusion that is classically associated with *P. falciparum* infection. Parasite metabolism also results in lactate production and release, further increasing a patient’s lactatemia. In addition, impaired clearance of lactate may occur owing to concurrent liver dysfunction.^42^^,^^43^ It is also possible that we underestimated cardiovascular dysfunction when using the pSOFA score if children with profound hypotension did not survive long enough to be admitted to the hospital.

The SICK score and LODS identified lower rates of MODS in pediatric CM patients. These scores offer the advantage of being designed for low-resource settings, are easily applicable, and perform well when predicting mortality. However, our attempt to organize these scores into system-based categories for the diagnosis of MODS proved unsuccessful. The SICK score is heavily reliant on vital sign abnormalities, which may not indicate dysfunction in a specific organ system. For example, tachycardia could represent cardiovascular dysfunction but may also be secondary to fever, anemia, respiratory distress, dehydration, or pain. The use of LODS as a MODS screening tool is limited as there are only three variables, two of which are distinctly neurologic in origin. Future work should focus on scoring systems that are easy to apply in resource-limited settings and target recognition of specific organ dysfunction in malaria patients.

Our study does have several limitations. This was a retrospective study conducted in a resource-limited setting. Despite having access to more laboratory capabilities than is typical in these settings, we had multiple missing values, most notably creatinine and bilirubin (Supplemental Table 5). Several missing data points are due to the rapid clinical decline and death of patients, but most of our missing data were due to lack of testing strips or reagents. Consequently, there is a strong possibility that we underestimated the prevalence of MODS or the severity of MODS in our cohort. This is especially true when utilizing the pSOFA score, which requires bilirubin, a laboratory value we did not obtain in 80 patients (40%) included in this study.

In addition, both respiratory dysfunction and renal dysfunction were potentially underrecognized in our cohort. Scoring systems such as PELOD-2 and pSOFA were developed and validated in high-resource settings. Owing to the absence of emergency airway management skills and resources in many malaria-endemic areas, a child with profound hypercapnia or hypoxemia would likely die prior to arrival or admission to the hospital. The use of laboratory values such as an arterial blood gas or the use of ventilator requirements is not feasible or applicable in this environment. Further work is needed to establish a more practical approach to evaluate the contribution of respiratory failure in this population. Similarly, these scoring systems use creatinine cutoffs based on data obtained from children in high-resource settings, an important limitation as serum creatinine levels are affected by nutritional status and muscle mass.^44^^,^^45^ Using the provided cutoffs, we likely underestimated AKI because of the risk of undernutrition in our cohort of Malawian children. Notably, PELOD-2 and pSOFA do not incorporate urine output criteria, the alternate recommended way to evaluate renal dysfunction. Monitoring the change in creatinine values when available or including urine output measurements as recommended by the Kidney Disease Improving Global Guidelines are promising potentials for evaluation of renal dysfunction in resource-limited settings.^46^

Lastly, it is possible that the prevalence of MODS was overestimated in our study. The presence of coma is part of the diagnostic criteria for CM. Therefore, all children had neurologic involvement. Multiple organ dysfunction syndrome could also be overestimated in this cohort owing to specific abnormalities related to the *P. falciparum* infection itself. Beyond elevated lactate, thrombocytopenia is regularly identified in children with malaria because of several mechanisms including immune-mediated destruction, splenic sequestration, bone marrow suppression, and consumption in the microvasculature.^47^^,^^48^ Unconjugated hyperbilirubinemia is also common in *P. falciparum* infections owing to hemolysis of both parasitized and unparasitized erythrocytes.^49^^–^^51^ In these patients, thrombocytopenia may not be the best marker of hematologic organ dysfunction, and hyperbilirubinemia may be a poor marker of liver dysfunction. Point-of-care tests are increasingly used in resource-challenged settings. Establishing which laboratory parameters are of most use in identifying children with severe malaria and concomitant MODS is imperative and will help to focus development efforts on affordable, feasible, and robust assays.

Multiple organ dysfunction syndrome was identified in nearly all children with CM. Although these results may not be generalizable to children with other types of severe malaria without further investigations, it is important to recognize the association of MODS and increased mortality in pediatric CM. Limitations in the currently available scoring systems may lead to over- as well as under-recognition of MODS in these patients. The development of a novel scoring system that rapidly and accurately identifies organ dysfunction in children across the spectrum of severe malaria manifestations would enhance clinical decision-making and facilitate effective resource allocation.

## Supplemental Materials

10.4269/ajtmh.24-0303 Supplemental Materials
