# Supplementary material for: Multiple Organ Dysfunction Syndrome in Malawian Children with Cerebral Malaria
Source: Am J Trop Med Hyg. 2024 Oct 8;111(6):1223–9. doi: 10.4269/ajtmh.24-0303 (PMC11619499; doi:10.4269/ajtmh.24-0303)
Supplement: Supplemental Materials [file tpmd240303.SD1.pdf]

Supplemental Tables

Supplemental Table 1. Pediatric Logistic Organ Dysfunction-2 (PELOD-2) Score with study modifications

Organ Dysfunction and Variables	Points by Severity Levels						
	0	1	2	3	4	5	6
**Neurologic**							
**Glasgow Coma Score**	≥ 11	5-10			3-4		
**Or**							
**Blantyre Coma Score**[α]	4-5	1-3			0		
**Pupillary reaction**	Both reactive					Both fixed	
**Cardiovascular**							
**Lactatemia (mmol/L)**	< 5.0	5.0-10.9			≥ 11		
**Mean arterial pressure (mmHg)**							
0 to <1 month	≥ 46		31-45	17-30			≤ 16
1 – 11 months	≥ 55		39-54	25-38			≤ 24
12 – 23 months	≥ 60		44-59	31-43			≤ 30
24 – 59 months	≥ 62		46-61	32-44			≤ 31
60 – 143 months	≥ 65		49-64	36-48			≤ 35
≥ 144 months	≥ 67		52-66	38-51			≤ 37
**Renal**							
**Creatinine (mg/dL)**[β]							
0 to <1 month	≤ 0.78		≥ 0.79				
1 – 11 months	≤ 0.25		≥ 0.26				
12 – 23 months	≤ 0.38		≥ 0.39				
24 – 59 months	≤ 0.57		≥ 0.58				
60 – 143 months	≤ 0.66		≥ 0.67				
≥ 144 months	≤ 1.04		≥ 1.05				
**Respiratory**							
**SpO_2 / FiO_2** [χ]	≥ 100		≤ 100				
**PaCO2 (mmHg)**	≤ 58	59-94		≥ 95			
**Invasive Ventilation**	No			Yes			
**Hematologic**							
**WBC Count (x10^9/L)**	> 2		≤ 2				
**Platelets (x10^9/L)**	≥ 142	77-141	≤ 76				

mmol = millimole; L = liter; mmHg = millimeters of mercury; mg = milligram; dL = deciliter; SpO_2 = oxygen saturation; FiO2 = fraction of inspired oxygen; PaCO2 = partial pressure of carbon dioxide; WBC = white blood cell

The following adjustments were made in this study: [α] Glasgow Coma Score converted into clinically equivalent Blantyre Coma Score;[26,31] Creatinine converted from μmol/L to mg/dL; SpO_2/FiO2 ratio applied instead of PaO2/FiO2 ratio [52,53]

Supplemental Table 2. Pediatric Sequential Organ Failure Assessment (pSOFA) Score with study modifications

Organ Dysfunction and Variables	Points by Severity Levels				
	0	1	2	3	4
**Respiratory**					
**PaO_2:FiO_2**	≥400	300-399	200-299	100-199 with respiratory support	<100 with respiratory support
**Or**					
**SpO_2:FiO_2**	≥292	264-291	221-264	148-220 with respiratory support	<148 with respiratory support
**Coagulation**					
**Platelet count x 10^3/µL**	≥150	100-149	50-99	20-49	<20
**Hepatic**					
**Bilirubin mg/dL**	<1.2	1.2 - 1.9	2.0 - 5.9	6.0 - 11.9	>12.0
**Cardiovascular**					
**Mean arterial pressure (mmHg)**			Dopamine hydrochloride ≤5 or dobutamine hydrochloride (any)	Dopamine hydrochloride >5 or epinephrine ≤0.1 or norepinephrine bitartrate ≤0.1	Dopamine hydrochloride >15 or epinephrine >0.1 or norepinephrine bitartrate >0.1
<1 months	≥46	<46			
1-11 months	≥55	<55			
12-23 months	≥60	<60			
24-59 months	≥62	<62			
60-143 month	≥65	<65			
144-216 months	≥67	<67			
>216 months	≥70	<70			
**Neurologic**					
**Glasgow Coma Score**	15	13–14	10-12	6-9	<6
**Or**					
**Blantyre Coma Score$^{\alpha}$**	5	4	3	1-2	0
**Renal**					
**Creatinine mg/dL**					
<1 months	<0.8	0.8-0.9	1.0-1.1	1.2-1.5	≥1.6
1-11 months	<0.3	0.3-0.4	0.5-0.7	0.8-1.1	≥1.2
12-23 months	<0.4	0.4-0.5	0.6-1.0	1.1-1.4	≥1.5
24-59 months	<0.6	0.6-0.8	0.9-1.5	1.6-2.2	≥2.3
60-143 month	<0.7	0.7-1.0	1.1-1.7	1.8-2.5	≥2.6
144-216 months	<1.0	1.0-1.6	1.7-2.8	2.9-4.1	≥4.2
>216 months	<1.2	1.2-1.9	2.0-3.4	3.5-4.9	≥5

PaO_2 = partial pressure of oxygen; FiO_2 = fraction of inspired oxygen; SpO_2 = peripheral capillary oxygen saturation; µL = microliter; mg = milligram; dL = deciliter; mmHg = millimeters of mercury,

The following adjustments were made for this study: $^{\alpha}$Glasgow Coma Score converted into clinically equivalent Blantyre Coma Score

Supplemental Table 3. Signs of Inflammation in Children that Can Kill (SICK) Score with study modifications

Organ Dysfunction and Variables	Score	
	0 points	1 point
Temperature	36°C -38°C	<36°C or >38°C
Cardiovascular[α]		
Heart Rate	Infant ≤160 per minute<br>Child ≤150 per minute	Infant >160 per minute<br>Child >150 per minute
Systolic blood pressure	Infant ≥65 mmHg<br>Child ≥75 mmHg	Infant <65 mmHg<br>Child <75 mmHg
Capillary refill time	<3 seconds	≥3 seconds
Respiratory[α]		
Respiratory rate	Infant ≤60 per minute<br>Child ≤ 50 per minute	Infant >60 per minute<br>Child > 50 per minute
SpO_2	≥90%	<90%
Neurologic[α]		
Alert, Voice, Pain, Unresponsive (AVPU)<br><br>Or<br><br>Blantyre Coma Score[β]	A	V, P, or U
	5	0-4

C = Celsius; mmHg = millimeters of mercury; SpO_2 = oxygen saturation.

The following adjustments were made in this study: [α]Scoring system variables were divided into organ specific categories; [β]AVPU converted into clinically equivalent Blantyre Coma Score

Supplemental Table 4. The Lambaréné Organ Dysfunction Score with study modifications

Organ Dysfunction and Variables	Score	
	0 points	1 point
Neurologic$^{\alpha}$		
Coma<br>Blantyre Coma Score ≤ 2	No	Yes
Prostration<br>Unable to breastfeed, sit, stand, or walk independently (age dependent)	No	Yes
Respiratory$^{\alpha}$		
Deep Breathing	No	Yes

The following adjustment was made in this study: $^{\alpha}$Scoring system variables were divided into organ specific categories

Supplemental Table 5. Missing variables for each scoring system

**Variable**	**Total Missing* (%)**
Age (months)	0 (0)
PELOD-2	
Blantyre Coma Score	0 (0)
Pupillary Reaction	0 (0)
Lactate	3 (1.5)
Systolic blood pressure	0 (0)
Diastolic blood pressure	0 (0)
Creatinine	26 (13)
Oxygen saturation	0 (0)
Amount of supplemental oxygen (FiO2)	0 (0)
PaCO2	60 (30)
Mechanical ventilation	0 (0)
White blood cell count	4 (2)
Platelet count	4 (2)
pSOFA	
Oxygen saturation	0 (0)
Amount of supplemental oxygen (FiO2)	0 (0)
Platelet count	4 (2)
Total bilirubin	80 (40)
Systolic blood pressure	0 (0)
Diastolic blood pressure	0 (0)
Blantyre Coma Score	0 (0)
Creatinine	26 (13)
SICK	
Temperature	0 (0)
Heart Rate	0 (0)
Systolic blood pressure	0 (0)
Diastolic blood pressure	0 (0)
Capillary refill time	0 (0)
Respiratory rate	1 (0.5)
Oxygen saturation	0 (0)
Reactive to pain	0 (0)
LODS	
Coma	0 (0)
Prostration	0 (0)
Deep breathing	0 (0)

PELOD-2 = Pediatric Logistic Organ Dysfunction-2; FiO2 = fraction of inspired oxygen; PaCO2 = partial pressure of carbon dioxide; pSOFA = Pediatric Sequential Organ Failure Assessment; SICK = Signs of Inflammation in Children that Can Kill; LODS = Lambaréné Organ Dysfunction Score

*N = 199
